# Ubiquitin and ubiquitin-like proteins in HPV-driven carcinogenesis

**DOI:** 10.1038/s41388-025-03310-6

**Published:** 2025-02-26

**Authors:** Louisa M. Wootton, Ethan L. Morgan

**Affiliations:** School of Life Sciences, University of Sussex, Brighton, UK

**Keywords:** Tumour virus infections, Virology

## Abstract

Persistent infection with high-risk (HR) human papillomaviruses (HPVs) is responsible for approximately 5% of cancer cases worldwide, including a growing number of oropharyngeal and anogenital cancers. The major HPV oncoproteins, E6 and E7, act together to manipulate cellular pathways involved in the regulation of proliferation, the cell cycle and cell survival, ultimately driving malignant transformation. Protein ubiquitination and the ubiquitin proteasome system (UPS) is often deregulated upon viral infection and in oncogenesis. HPV E6 and E7 interact with and disrupt multiple components of the ubiquitination machinery to promote viral persistence, which can also result in cellular transformation and the formation of tumours. This review highlights the ways in which HPV manipulates protein ubiquitination and the ubiquitin-like protein pathways and how this contributes to tumour development. Furthermore, we discuss how understanding the interactions between HPV and the protein ubiquitination could lead to novel therapeutic targets that are of urgent need in HPV+ carcinomas.

## Introduction

*Papillomaviridae* is a family of small, double-stranded, non-enveloped icosahedral DNA viruses containing a genome of around 8kb [1]. Over 400 genotypes have been identified to date (referred to as types), of which over 200 types are human papillomaviruses (HPV) [2, 3]. HPVs infect host keratinocytes in the stratified squamous epithelia, and most HPVs share a common genome organization and express a similar set of viral genes [1, 4]. Persistent HPV infection initiates and is maintained in basal keratinocytes, with keratinocyte differentiation enabling viral genome replication and the production of infectious virions [5–7]. Clinically, HPVs can be categorised as low-risk (LR) or high-risk (HR) based on their association with the development of malignant lesions. Low-risk HPVs are generally associated with benign warts, whereas persistent infection with HR-HPV, particularly HPV16 and HPV18, is associated with a number of cancers, most notably cervical cancer [8]. As the most common sexually transmitted infection, HPV is responsible for around 5% of all cancer cases worldwide [8]. The association between HR-HPV infection and cancer was established over 40 years ago, when HPV16 DNA was found to be highly prevalent in cervical carcinoma biopsies; however, only 10–20% of infections persist and result in malignant development [9].

To date, 15 HR-HPV types have been identified; HPV16, 18, 33, 35, 31, 39, 45, 51, 52, 56, 58, 59, 68, 73, and 82. Collectively, these are responsible for >99.7% of cervical cancer cases in women, the 4th most common cancer in women worldwide, with 55% being HPV16-positive and 15% HPV18-positive. Furthermore, HPV has also been associated as an aetiological agent in four other ano-genital cancers: anal, vaginal, vulvar and penile cancer [2]. Additionally, cases of HPV-associated oropharyngeal cancer have been increasing, mostly in men [8].

The HPV viral oncoproteins E5, E6 and E7 are critical drivers of carcinogenesis in HPV-associated cancers [10]. Together the HPV oncoproteins act to provide an environment which favours viral replication, by prolonging the proliferation and delaying cellular differentiation of keratinocytes. The most well studied mechanisms of which are the degradation of the tumour suppressor retinoblastoma protein (pRb) by HPV E7, bypassing the G1/S restriction and causing aberrant cell cycle entry [11], and the HPV E6-mediated degradation of the p53 tumour suppressor, allowing cells to undergo persistent, aberrant proliferation [2, 12]. The oncogenic mechanisms of HPV E5 are less well studied, but it plays a supportive role in promoting proliferation and delaying differential via the EGFR signalling pathway [13, 14].

As mentioned above, the two most well studied functions of HPV E6 and E7, namely the proteasomal degradation of the tumour suppressor proteins p53 and pRb, require the co-opting of the host ubiquitin-proteasome system (UPS). The UPS regulates almost all cellular processes, such as DNA repair, the cell cycle, and the immune response, therefore playing a critical role in cellular homoeostasis. Moreover, a growing number of oncogenes and tumour suppressors have been shown to be heavily regulated by ubiquitination, thus providing an attractive therapeutic target in oncology [15].

In this review, we will describe the interactions between the HPV oncoproteins and the ubiquitin machinery, as well as the related ubiquitin-like proteins (ULP). We will discuss their role in HPV-associated carcinogenesis and provide an insight into potential clinical implications and how targeting these pathways may provide novel therapeutic targets for the treatment of HPV-associated cancers.

## Protein ubiquitination

**Fig. 1:**
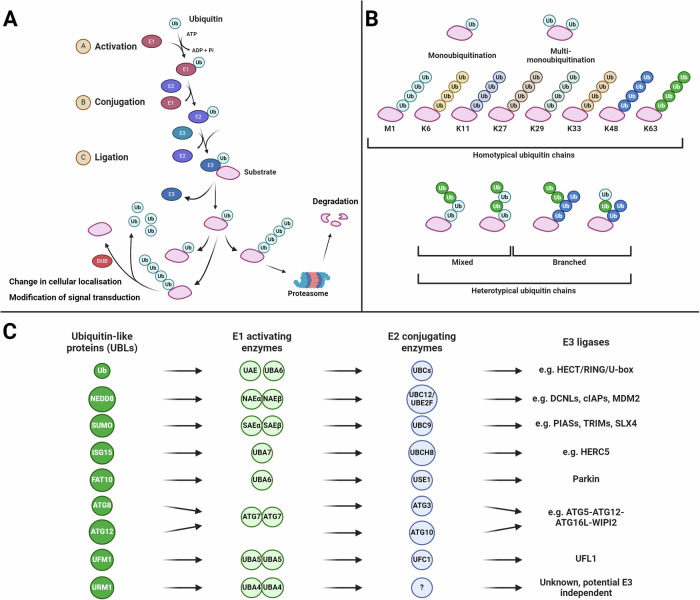
Ubiquitination pathway, ubiquitination chain types and different ubiquitin-like proteins (UBL). **A** A schematic of the ubiquitin enzymatic cascade. Ubiquitin is added to protein substrates via the E1-E2-E3 enzymatic cascade. E1 E1-activating enzyme, E2 E2-conjugating enzyme, E3 E3 ubiquitin ligase, DUB deubiquitinating enzyme. **B** Schematic of the multiple different types of ubiquitination: mono- and multi-monoubiquitination, the 8 types of homotypic ubiquitination (M1, K6, K11, K27, K29, K33, K48, K63), and the two types of heterotypic ubiquitination, mixed and branched. **C** Nine classes of ubiquitin-like proteins (UBL) and eight E1 activating enzymes have been discovered in humans. UBLs and their associated E1 and E2 enzyme are structurally related, but are unique to each pathway. Ubiquitin can, however, use two E1 proteins, UAE and UBA6. Ub ubiquitin, UAE ubiquitin activating enzyme, NAE NEDD8-activating enzyme, SAE SUMO-activating enzyme. Figure created with BioRender.com.

Ubiquitin is a small, highly conserved, ubiquitously expressed protein found in all eukaryotic species [16, 17]. Protein ubiquitination is the mechanism of ubiquitin conjugation to protein substrates, which regulates many cellular processes including DNA repair, the cell cycle, and cellular differentiation and development [18]. Protein ubiquitination involves a three-step enzymatic cascade (Fig. 1A). Firstly, the activation of ubiquitin by ubiquitin-activating enzyme (E1), the subsequent transfer of ubiquitin to a ubiquitin conjugating enzyme (E2), and then conjugation to a protein substrate via a ubiquitin E3 ligase, which are substrate specific [19].

To date, studies have identified two E1 enzymes for ubiquitination, 40 E2 enzymes and over 700 E3 ligases. Protein ubiquitination can have several consequences, depending on the type of ubiquitin linkage and chain length: the activation or deactivation of intracellular signalling, a change in protein localisation, or in protein degradation [18]. Ubiquitin contains 76 amino acids, which includes seven lysine residues that are the sites of ubiquitin linkage. Ubiquitin linkages at any of these seven lysine residues (lysine 6 (K6), K11, K27, K33, K48, and K63; Fig. 1B) via their C-terminus [20–22]. Furthermore, ubiquitin can be directly attached to the N-terminal methionine, forming linear polyubiquitin chains [18]. Ubiquitination can involve one ubiquitin molecule (monoubiquitination) or multiple ubiquitin molecules (polyubiquitination); polyubiquitin chains can be homotypic (chains containing the same linkage e.g. K48-polyubiquitin chains), or heterotypic (chains contain multiple types of linkages), which can be subcategorised as mixed (multiple ubiquitin linkages in a chain) or branched (lysine residues modified by more than one different linkage). Mixed chains consist of ubiquitin subunits that are modified on only a single acceptor site. Additionally, ubiquitin can also be phosphorylated or acetylated [22]. Finally, more recent studies have demonstrated that in addition to protein ubiquitination, lipids, sugars and nucleic acids can also be ubiquitinated [23]. Ubiquitination is a reversable post-translational modification and as such, there are over 100 deubiquitinating enzymes which cleave ubiquitin from target substrates; these enzymes also have an essential role in the regulation of ubiquitination and its effects, as well as the recycling of ubiquitin molecules28. Severn families of deubiquitinase enzymes have been identified to date; ubiquitin C-terminal hydrolases (UCHs), ubiquitin-specific proteases (USPs), ovarian tumour proteases (OTUs), JAB1/MPN/MOV34 metalloenzymes (JAMMs), the Machado-Josephin domain superfamily (MJD), the MINDY family, and the ZUFSP family members [23, 24]. Deubiquitinases can be either highly promiscuous (e.g. USP2) or highly specific (e.g. OTULIN) in regard to substrate specificity. The level of complexity in the ubiquitin system highlights its critical regulatory role in cellular homoeostasis.

## The ubiquitin proteasome system

Protein ubiquitination was originally identified as an ATP-dependent proteolytic system in mammalian cell extracts [25, 26]. The most well characterised ubiquitin linkage type, K48-polyubiquitination, functions as proteolytic signal; a chain of at least four K48-linked ubiquitin molecules can then be recognised the 26S proteasome [27]. This large molecular machine contains a central proteolytic core made of four ring structures, flanked by two cylinders. Once inside, proteins are rapidly degraded into small peptides and ubiquitin molecules are cleaved prior to degradation and are recovered for further use [28].

The UPS is a highly conserved pathway, from yeast to mammals; it is an essential pathway for cells to be able to regulate intracellular protein concentration and remove misfolded proteins, avoiding toxic build-up [28]. It regulates multiple cellular processes, such as the cell cycle, as the timely destruction of certain proteins is vital for controlled cell division [29]. Therefore, the deregulation of the UPS can have significant consequences in pathology, such as in viral infection and cancer development [29, 30].

## Ubiquitin-like proteins

In addition to ubiquitin, the human genome contains several proteins that share a similar three-dimensional structure, a so-called ‘β-grasp’ protein fold (a five strand beta sheet surrounding an alpha helix), and are thus called ubiquitin-like proteins (UBLs) (Fig. 1C) [31]. These proteins are conjugated to proteins via an enzymatic cascade that is similar to that of ubiquitin, and several components of these pathways are either involved in, or are highly related to, components of the ubiquitination pathway [31]. To date, 17 proteins have been classified as UBLs, in 8 distinct families (Fig. 1C): NEDD8, SUMO (SUMO1-4), ISG15, FAT10, ATG8 (LC3A, LC3B, LC3B2, LC3C, GABARAP, GABARAPL1, GABARAPL2), ATG12, UFM1 and URM1. Since their discovery, substrates for many of these UBLs and their associated functions have been uncovered. Conjugation of NEDD8, termed NEDDylation, plays a critical role in the activation of cullin-based E3 ubiquitin ligases [32]. SUMO conjugation, termed SUMOylation, regulates many cellular processes, such as protein stability, gene transcription and cell cycle progression [33]. Both ISG15 conjugation (ISGylation) and FAT10 conjugation play an important role in anti-viral immunity [34, 35]. ATG8 and ATG12 conjugation plays an essential role in autophagic vesicle formation [36]. As for UFM1 (UFMylation) and URM1 (URMylation), relatively little is known about their specific substrates and biological roles, but they may be involved in the function of the ER and in the cellular stress response, respectively [37, 38].

## HPV and protein ubiquitination

As previously mentioned, protein ubiquitination and the UPS has been identified as dysregulated in a number of pathologies, including viral infection and malignant development [39, 40]. Notably, the hallmark function of the HPV oncoproteins E6 and E7 is the binding of the tumour suppressor proteins p53 and pRb, respectively, promoting their ubiquitination and proteasomal degradation [41, 42]. Furthermore, many of the target proteins of HPV E6 and E7 are directed for proteasome-mediated degradation [43]. Thus, the HPV oncoproteins can manipulate the ubiquitin system to drive cell proliferation and prevent apoptosis, ultimately promoting cellular transformation.

## Protein ubiquitination in HPV-associated oncogenesis

### E3 ubiquitin ligases

**Fig. 2:**
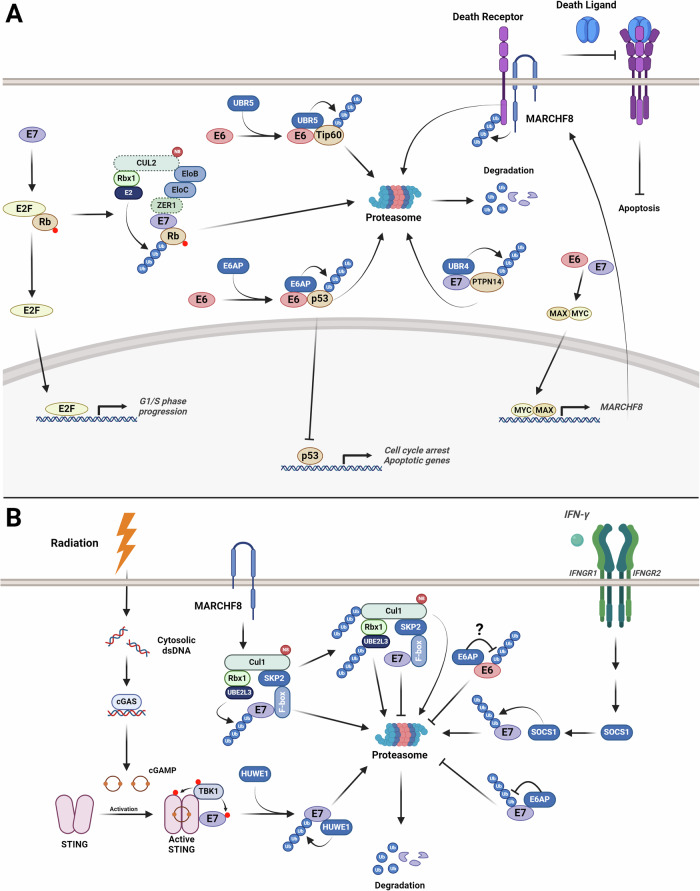
HPV oncoproteins and E3 ligases. **A** Interactions of the HPV oncoproteins with E3 ligases. HPV E6 interacts with E6AP to promote the proteasomal degradation of p53 and UBR5 to promote the degradation of Tip60. HPV E7 interacts with a NEDDylated, CUL2-dependent E3 ligase complex to promote the proteasomal degradation of pRb. Both E6 and E7 promote the expression of MARCHF8, preventing apoptosis via the degradation of death receptors such as TRAIL-1 and TRAIL-2. Dashed boarder on CUL2 and ZER1 indicates that the interaction has only been observed for HPV16 E7. **B** Regulation of the HPV oncoproteins by E3 ligases. HPV E6 is potentially negatively regulated by E6AP. HPV E7 interacts with a CUL-SKP2 complex that promotes the ubiquitination and proteasomal degradation of E7. Radiation treatment promotes the phosphorylation of E7 via TBK1 and the subsequent binding of HUWE1 and E7 ubiquitination and proteasomal degradation. IFNγ signalling promotes SOCS1 binding to E7 and its ubiquitination and proteasomal degradation. E6AP can promote the stability of E7 via reduced E7 ubiquitination. Figure created with BioRender.com.

Many tumour viruses manipulate the UPS to promote viral persistence, and this can result in cellular transformation [44, 45]. The two best studied functions of the HPV oncoproteins are the promotion and subsequent degradation of the tumour suppressor proteins p53 and pRb, respectively. HPV E6, via the presence of an LXXLL binding motif, forms a stable, heterotrimeric complex comprising of E6, E6AP and p53; this stimulates E6AP E3 ligase activity, promoting p53 polyubiquitination and proteasomal degradation (Fig. 2A) [41, 46]. The degradation of p53 results in uncontrolled cell proliferation and the inhibition of apoptosis; interestingly, although low-risk E6 proteins can bind to E6AP, they do not interact with or induce the degradation of p53 [47].

In addition to p53, several other targets of the E6-E6AP complex have been identified in recent years. HR-HPV E6 can promote the E6AP-dependent proteasomal degradation of Na + /H+ Exchanger Regulatory Factor 1 (NHERF1), a scaffolding protein involved in the trafficking and signalling of G protein-coupled receptors (GPCRs) [48, 49]. Subsequent studies demonstrated that degradation of NHERF1 promoted PI3K/AKT and WNT signalling, promoting oncogenesis in cervical cancer cells [48, 49]. Recently, E6 has also been shown to induce chromosomal instability by inducing the degradation of the mitotic kinesin CENP-E; this degradation requires E6AP activity [50].

In addition to the to its well-studied interactions with E6AP, HPV E6 can also interact with or modulate the function of other E3 ligases to promote oncogenesis. Ubiquitin protein ligase E3 component n-recognin 5 (UBR5; also called EDD1) is a HECT-domain containing E3 ubiquitin ligase [51]. In HPV+ cervical cancer, E6 destabilises the histone acetyltransferase TIP60, which has a major role in maintaining genomic stability (Fig. 2A) [52]. Degradation of TIP60 by HPV is essential for p53 degradation, as TIP60 functions as a repressor of the HPV promoter and thus prevents E6 expression [53]. Subsequent studies revealed that UBR5 is a TIP60-binding protein and promotes its ubiquitination and proteasomal degradation in a HPV E6-dependent manner [54]. Interestingly, prior work had revealed that E6 interacts with UBR5, negatively regulating E6 expression and the function of the E6/E6AP complex, suggesting a complex relationship between HPV E6 and URB5 [55].

A recent study demonstrated that HPV E6 (and potentially E7) can promote the expression of the E3 ligase MARCHF8 via the MAX/MYC transcription factors [56]. This leads to the downregulation of the death receptors FAS, TRAIL-R1 and TRAIL-R2 in HPV + HNC cells, preventing apoptosis and promoting tumour growth in vivo [56].

HPV E7 promotes S-phase entry in infected cells via pRb and the related pocket proteins p107 and p130 via the conserved LXCXE motif located within its Conserved Region (CR)2 domain; this induces dysregulation of E2F transcription factors, promoting expression of genes involved in S-phase progression [57, 58]. The degradation of Rb family members by HPV16 E7 requires a CUL2-containing E3 ligase complex consisting of CUL2, Rbx1 and Elongin B and C, where E7 binds to the Elongin C subunit (Fig. 2A) [59–61]. The substrate specificity factor ZER1 is required for the binding of HPV16 E7 to CUL2, and therefore the subsequent degradation of pRb [62], and contributes to the oncogenic properties of HPV16 E7 [60]. However, this interaction and activity may not be consistent across different HPV types, as no association between HPV18 E7 and CUL2 was observed [60]. Furthermore, a study demonstrated that HPV16 E7 mutants that cannot bind CUL2 can still degrade pRb [63]; together, these studies suggest that other E3 ligases may contribute to HR-HPV E7-mediated degradation of pRb across different HPV types.

Few other substrates for the E7-CUL2 complex have been identified. The cytidine deaminase APOBEC3A (A3A) is upregulated in HPV+ cancers, and this is due to HPV E7 mediated stabilisation [64]. HPV16 E7 and CUL2 were able to bind to A3A; however, HPV16 E7 did not bind directly to A3A and may require the interaction with CUL2 to prevent A3A degradation [64].

HR-HPV E7 can also bind to the E3 ligase UBR4 (also called p600), and a major target of this interaction is the protein tyrosine phosphatase PTPN14 [65–67]. E7 recruits UBR4 to target PTPN14 for proteasomal degradation and E7 mutants that cannot bind UBR4 and deficient in their transforming ability. PTPN14 is a negative regulator of YAP1, an oncogenic transcription factor that is regulated by the Hippo signalling pathway [68]; PTPN14 can also activate the Hippo pathway via interaction with LATS1 [69]. Given the demonstrated importance of YAP/TAZ and the inactivation of Hippo signalling in HPV+ cancers [70–73], the role of E7-mediated PTPN14 via co-opting the UBR4 E3 ligase likely has a critical function in E7-mediated oncogenesis [74].

HPV E7 has also been found to interact with RNF168, a RING-domain E3 ubiquitin ligase which ubiquitinates H2AX, H2AZ and H2A and plays essential in the repair of DNA double strand breaks (DSBs) [75]. RNF168 is highly expressed in HPV+ cervical cancer and HNSCC HPV E7 limits the function of RNF168 at DSBs, favouring DNA repair by homology-directed recombination repair (HDR).

Together, these studies demonstrate the manipulation of host ubiquitination by the HPV oncoproteins; however, several E3 ligases play a role in HPV+ cancer progression due to genetic alterations observed in these cancers. The F-Box and WD Repeat Domain Containing 7 protein (FBXW7), a component of Skp1-Cullin1-F-box (SCF) complexes, has been shown to regulate cellular growth and act as a tumour suppressor in several cancers [76]. FBXW7 is often mutated at a greater frequency in HPV+ cancers when compared to HPV- cancers, with mutation frequencies of between 7% to 15% in cervical cancer [77, 78], ~10% in anal cancer [79], ~10% in vulvar cancer [80] and between 7.7–20% in HPV + HNSCC [81]. *FBXW7* mutations p.R479P and p.L443H were found to promote invasion, migration and proliferation of cervical cancer cells, suggesting that *FBXW7* mutations may be oncogenic in HPV+ cancers [82]. Whilst there are numerous studies highlighting that *FBXW7* mutations are enriched in HPV+ cancers, investigating the substrates and effects of these mutations are needed to provide a better understanding of their function. A key finding of the HNSCC TCGA study identified common mutations or deletions in the *TRAF3* gene in HPV + HNSCC [83, 84]. TRAF3, an E3 ligase, is a negative regulator of both canonical and non-canonical NFκB activity [85, 86]; mutations in *TRAF3*, or the loss of *TRAF3*, results in enhanced NFκB signalling and promoted HPV + HNSCC development [83, 86, 87].

## Deubiquitinating enzymes

**Fig. 3:**
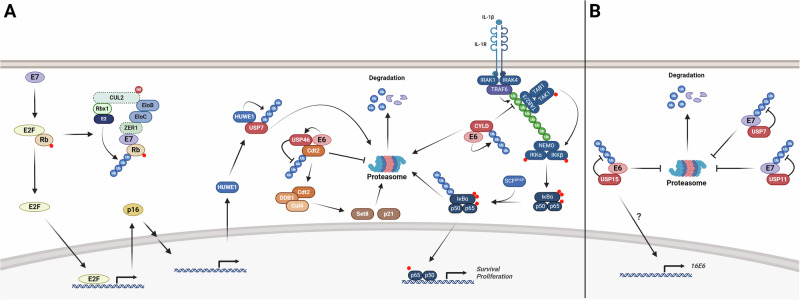
HPV oncoproteins and deubiquitinating enzymes. **A** Interactions of the HPV oncoproteins with deubiquitinating enzyme. HPV E6 interacts with CYLD to promote it’s proteasomal degradation and the activation of the NFκB pathway. HPV E6 also interacts with USP46, preventing the degradation of Cdt2, a component of the CRL4^Cdt2^ E3 ligase complex, resulting in the degradation of p21 and Set8. HPV E7 mediated p16 expression promotes the expression of HUWE1, which promotes E7 ubiquitination and proteasomal degradation. Dashed boarder on CUL2 and ZER1 indicates that the interaction has only been observed for HPV16 E7. **B** Regulation of the HPV oncoproteins by deubiquitinating enzymes. HPV E6 binds to USP15, which deubiquitinates E6, preventing its proteasomal degradation. HPV E7 can bind to both USP7 and USP11, which deubiquitinates E7 and prevent its proteasomal degradation. Figure created with BioRender.com.

As the regulation of ubiquitination can have an impact on both protein stability and the activation or termination of various signalling pathways involved in oncogenic processes, deubiquitinating enzymes are often deregulated in cancer [88]. As a consequence of this, deubiquitinating enzymes are becoming an attractive target in the oncology space, with several small molecule inhibitors entering pre-clinical and early phase clinical trials [88]. In HPV-associated cancers, several deubiquitinating enzymes having been shown to play oncogenic functions. Early work demonstrated that HPV E6 can drive the degradation of CYLD, a critical negative regulator of NFκB signalling [89]. NFκB is a pro-inflammatory signalling pathway that plays a role in many cancers, including squamous cell carcinomas such as cervical cancer and HNSCC [90–93]. In hypoxia, HPV E6 promotes the polyubiquitination of CYLD by an unknown mechanism, promoting hypoxia-induced NFκB signalling and cervical cancer cell proliferation (Fig. 3A) [89]. The HNSCC TCGA project also identified common mutations or deletions in the *CYLD* gene in HPV + HNSCC; as expected, mutations in *CYLD* result in enhanced NFκB signalling [84].

HPV E6 has also been shown to regulate the function of another USP; E6 forms a complex with USP46 in cervical cancers cells [94]. This promotes the interaction of USP46 and Cdt2, a component of the CRL4^Cdt2^ E3 ligase complex, promoting its stability (Fig. 3A) [94]. The stabilisation of Cdt2 is essential for the proliferation of HPV+ cervical cancer cells; further studies demonstrated that the Cdt2 target Set8, a histone methyltransferase, is reduced in cervical cancer and is a primary target of HPV E6-USP46 [94, 95].

Additionally, several deubiquitinating enzymes have been shown to play a role in therapeutic resistance in HPV-associated cancers. USP13, which is sits on chromosome 3q26 that is highly amplified in cervical cancer, plays a key role in regulating Mcl-1 expression in HPV+ cervical cancer cells [96]. Mcl-1 is a pro-survival protein that is often associated with resistance to BH3 mimetic treatment [97]; this study demonstrated that the depletion or inhibition of USP13 sensitises HPV+ cervical cancer cells to BH3-mimetics, a potential therapeutic approach in these cancers [98]. Two other deubiquitinating enzyme, OTUD1 and JOSD1, have also been shown to regulate Mcl-1 in cervical cancer, suggesting that the regulation of Mcl-1 ubiquitination may be essential for cervical cancer progression [99, 100].

Recently, a study demonstrated a novel mechanism of radiation sensitivity in HPV + HNSCC. p16, the clinically used surrogate for HPV positivity, is known to be involved in the response to DNA damage and can promote radiosensitivity [101, 102]. However, how p16 functions in HPV + HNSCC, where it is significantly upregulated, is unclear. Recent work has demonstrated that p16 promotes the ubiquitination and degradation of USP7, resulting in low expression of USP7 in HPV + HNSCC [103]. This is due to the p16-dependent transcriptional regulation of the E3 ligase HUWE1, which induces the polyubiquitination and subsequent proteasomal degradation of USP7 (Fig. 3A). The decreased expression of USP7 in HPV + HNSCC results in lower TRIP12 expression, decreasing homologous recombination and enhancing the response to radiotherapy [103]. Of particular interest, this study demonstrated that inhibition of USP7 could sensitise HPV- HNSCC cells to both radiation treatment and PARP inhibition, suggesting that combination therapies may be beneficial in these cancers [103].

## Regulation of HPV oncoproteins by the UPS

Both HPV E6 and E7 are short-lived, unstable proteins, with half-lives between 30 and 90 min [104, 105]. Each protein is stabilised in the presence of proteasome inhibitors, suggesting that ubiquitination and subsequent proteasomal degradation is a key regulator of the HPV oncoproteins [104]. Furthermore, both proteins are stabilised when in complex with cellular proteins. For example, E6 can be stabilized by heterodimerization with E6-AP through an acidic LXXLL motif [106] and both E6 and E7 can self-dimerise, forming stable homodimers [107, 108]. More recently, the HPV16 protein E6^E7 protein, a splice isoform protein in HPV16, can stabilise both E6 and E7 via interactions with the chaperone proteins HSP90 and GPR78 [109]. Together, these studies make it clear that the HPV oncoproteins are unstable and their protein expression is heavily regulated via protein-protein interactions and the UPS.

### E3 ubiquitin ligases

HPV E6 has been shown to be polyubiquitinated in cells [110]; however, little is known about the mechanisms of E6 ubiquitination and the E3 ligase responsible for is unclear. A recent study has shown that depletion of E6AP, the E6 binding protein that promotes p53 degradation, can stabilise E6 and decreased E6 polyubiquitination (Fig. 2A) [110]. However, several studies have shown contradictory results, demonstrating that E6AP stabilises HPV E6 [111, 112]. Therefore, direct evidence of E6AP promoting the ubiquitination of E6 and its effect on E6 stability requires further investigation. Recently, FBXO4 was identified as an E3 ligase that promotes HPV18 E6 ubiquitination and degradation in the absence of E6AP [113]. Further data demonstrated that FBXO4 also promoted the degradation of HPV11 E6 in E6AP-null cells, but not HPV16 E6 [113].

The mechanism of HPV E7 ubiquitination is clearer, with several E3 ligase implicated in promoting E7 polyubiquitination. As with HPV E6, HPV E7 is polyubiquitinated and degraded by the proteasome [114]. Interestingly, HPV16 E7 only has two lysine residues, and early studies demonstrated that neither lysine is required for E7 polyubiquitination; the mechanism of E7 ubiquitination is via the N terminus, termed N-terminal ubiquitination [114, 115].

Further analysis of E7 ubiquitination demonstrated that the E2 ubiquitin-conjugating enzyme UbcH7 (also called UBE2L3) and a Cullin-1 and Skp-2 containing E3 ubiquitin-ligase, a member of the family of SKP-CULLIN-F box ubiquitin ligase complexes (SCF), interact with E7 to promote its ubiquitination [116]. In vitro ubiquitination assays showed that UbcH7 is the specific E2 enzyme involved in the ubiquitination of E7; other E2 ligases, such as UbcH6 and UbcH5a, did not play a significant role in E7 ubiquitination. HPV E7 can interact with both CUL1 and SKP2, both in vitro and in vivo. The importance of SKP2 in regulating HPV E7 stability was supported by experiments in SKP2 knock out (KO) MEFs, suggesting that the CULl-SKP2 complex plays a significant role in E7 ubiquitination and proteolysis [116]. Highlighting the role of this complex in promoting E7 ubiquitination and proteasomal degradation, the E3 ligase MARCHF8, which is upregulated by HPV E6 and E7, binds to UbcH7 and CUL1 to promote their ubiquitination and proteasomal degradation, thereby preventing HPV16 E7 degradation [56, 117]. Therefore, HPV has evolved effective countermeasures to prevent the degradation of HPV E7 to promote the viral life cycle and subsequent cellular transformation.

Several other E3 ligases have also been implicated in E7 ubiquitination. Interferon γ (IFNγ), an antiviral cytokine, can induce E7 polyubiquitination HPV+ cervical cancer cells [118]. This is due to IFNγ-mediated induction of the E3 ligase suppressor of cytokine-signalling-1 (SOCS1), which promoted E7 polyubiquitination (Fig. 2A) [118]. However, E7 is still partially ubiquitinated in the presence of an enzymatically defective SOCS1, suggesting the involvement of other E3 ubiquitin ligases in the regulation of E7 polyubiquitination [118].

In response to radiation treatment, the stimulator of interferon genes (STING) is activated and promotes tumour cell death by promoting DNA damage stimulator of interferon genes [119]. In HPV+ cells, HPV E7 can bind to STING, promoting its degradation in an autophagy-dependent manner to avoid immune detection [120]. However, a recent report demonstrated that STING activation in response to radiation treatment or a STING agonist promotes STING dimerization and recruitment of the kinase TBK1, which subsequently phosphorylate both STING and STING-bound HPV E7 (Fig. 2A) [121]. This promoted recruitment of the E3 ligase HUWE1, which induces the polyubiquitination and proteasomal degradation of HPV E7, inhibiting cervical cancer cell proliferation [121].

Interestingly, a recent study has demonstrated that the E6 binding protein E6AP can also bind and stabilise HPV E7 [122]. E6AP can bind to E7, preventing its polyubiquitination and proteasomal degradation by an unknown mechanism, promoting HPV+ cervical cancer cell growth [122]. Of particular note, the co-expression of HPV E6 contributes to E6AP-mediated E7 stability, demonstrating the coordinated nature of the HPV oncoproteins in driving cellular transformation.

Of particular interest, both HPV E6 and E7 have been shown to bind to the proteasome via distinct mechanisms. HPV E6 can bind to multiple proteasomal subunits, particularly S4, S5a and S8 [123]. Notably, S5a is the major ubiquitin-accepting proteasome subunit and the interaction with HPV E6 requires E6AP [123]. Older work demonstrated that HPV E7 can also interact with the proteasome through its S4, and this enhances the ATPase activity of the S4 subunit; however, the biological function of this interaction is not clear [124]. These studies demonstrate the complex associations of the HPV oncoproteins with the proteasome that may have significant consequences in oncogenic development.

### Deubiquitinating enzymes

Whilst the ubiquitination of the HPV oncoproteins has been fairly well studied, particularly for HPV E7, the process of deubiquitination is less understood, with only a handful of deubiquitinating enzyme have been shown to regulate HPV oncoprotein stability. USP15 is a member of the Ubiquitin Specific Protease (USP) family of deubiquitinating enzymes (Fig. 3A) [125]. Tandem affinity purification of HPV16 E6 interacting proteins identified USP15 as a novel interacting protein of HPV16 E6; USP15 expression increased E6 stability and protein expression. Interestingly, USP15 also promoted E6 mRNA expression by an unknown mechanism. Subsequent studies confirmed this interaction and demonstrated that USP15 functioned as a deubiquitinating enzyme for HPV16 E6 and plays a key role in the HPV life cycle by antagonising the activation of the innate immune sensor RIG-I [126–128].

For HPV E7, USP11 has been shown to interact with HPV16 E7 and promote its stability to through its deubiquitination activity (Fig. 3A) [129]. This study demonstrated that USP11 depletion decreased the proliferation of HPV16+ cervical cancer cells, suggesting that USP11-mediated 16E7 stability is required for its oncogenicity. Recently, USP7, also known as HAUSP, has been shown to regulate HPV E7 stability [130]. USP7, originally identified as a binding partner of Herpes simplex virus 1 (HSV-1) ICP0, has previously been implicated as a pro-viral factor in a number of oncogenic viruses, including Kaposi’s Sarcoma Herpesvirus (KSHV) and Epstein-Barr virus (EBV) [131]. USP7 was demonstrated to regulate the stability of HPV16 E7 and inhibition of USP7 reduced the proliferation, migration and invasion of HPV16+ cervical cancer cells [130]. Additionally, HPV E7 has been shown to interact with a number of other deubiquitinating enzymes, including USP26, USP29 and USP33, but the function of these interactions is currently not known [128].

## HPV and ubiquitin-like proteins

As discussed, several ubiquitin-like proteins (UBLs) exist in the human genome that also play a key role in the regulation of multiple cellular process [132]. Like ubiquitin, several UBLs covalently modify their target proteins through an enzymatic cascade similar to that of ubiquitination [132]. Whilst a number of UBLs have been found, only a few have been identified as having an important role in HPV-associated disease.

### ISG15

ISG15, an interferon-stimulated, 15 kDa gene product, was the first of a number of ubiquitin-like proteins to be identified as a protein modifier [34]. Whilst the function of ISG15 is not well understood, it is known to play a key role in anti-viral immunity [34]. In HPV+ cervical cancer, HPV E7 can prevent the expression of the ISG15-sepcific E2 enzyme UBCH8 by upregulating the expression of UHRF1, thereby promoting *UBE2L6* gene promoter methylation [133]. However, the role of ISG15 in HPV+ cervical cancer progression is not clear; one study demonstrated that ISG15 depletion reduces proliferation in HPV+ cervical cancer cells, whilst another demonstrated that ISG15 over-expression reduces HPV+ cervical cancer cells growth in vivo [134, 135]. Therefore, the role of ISG15 in HPV+ cervical cancer, and other HPV+ cancers, is unclear and requires further investigation.

### SUMO

SUMO, a 12 kDa ubiquitin-like protein, is conjugated to cellular proteins in a process termed SUMOylation. Several types of SUMO exist, with SUMO1 and SUMO2/3 forming SUMO conjugates on protein substrates. SUMOylation has broad ranging effects, including the regulation of nuclear transport, transcription and the stress response [33]. SUMOylation has been shown to play a role in viral infections, with many viral proteins being modified by SUMO as an anti-viral mechanism [136]. +Thus, many viruses manipulate the SUMOylation pathway to prevent this, and this can subsequently lead to viral persistence and cell survival [136].

The interaction of HPV with the SUMOylation pathway is not well understood and conflicting data exists within the literature. HPV E6 was shown to promote the degradation of the SUMO E2 enzyme UBC9, reducing global SUMOylation rates in keratinocytes [137]; however, more recent work has demonstrated that UBC9, SUMO1 and SUMO2/3 are over expressed in HPV+ cervical cancer and HNSCC [138]. The authors demonstrated that expression of E6 and E7 promoted UBC9 expression by preventing its degradation by autophagy, suggesting that the combination of E6 and E7 is required to promote UBC9 expression and enhanced SUMOylation. In line with this, the expression of UBC9 has also been shown to be a potential diagnostic marker in cervical cancer [139].

### NEDD8

Ubiquitin-like protein neural precursor cell expressed, developmentally downregulated 8 (NEDD8) is a 9 kDa protein [32]. A primary target for NEDDylation, the conjugation of NEDD8 proteins on target substrates, is the Cullin-RING ligase family (CRL). These E3 ligase complexes require NEDD8 modification of their C-terminus in order to become activated^14-^. This includes CUL2, a ubiquitin ligase that interacts with HPV E7; indeed, E7 preferentially interacts with the NEDDylated form of CUL2 [59]. This suggests that NEDD8 may be a potential therapeutic target in HPV+ cancers by preventing CRL activation and the interaction of E7 with pRb; as such, inhibition of the NEDD8 activating enzyme (NAE) with MLN4924 prevents E7-mediated pRb degradation [60]. Furthermore, MLN4924 supressed HPV+ cervical cancer growth, both in vitro and in vivo [140, 141]; however, whether these effects are specific to HPV+ cervical cancer is unclear.

## Clinical implications

Alterations in ubiquitin and ubiquitin-like protein signalling are commonly observed not only in HPV-associated cancers but across many different cancer types and other pathologies, and targeting these signalling pathways, particularly ubiquitination, is becoming an attractive target in cancer therapy [142]. For example, several small molecule inhibitors targeting protein ubiquitination are in clinical trials for various cancers [143]. In HPV+ cancer, proteasome inhibitors have demonstrated good results in pre-clinical studies, but poor efficacy in clinical trials for both cervical cancer and HNSCC [144]. Furthermore, inhibition of inhibitor of apoptosis proteins (IAPs), cellular proteins that inhibit apoptosis and drive pro-inflammatory NFκB signalling [145], recent pre-clinical studies have demonstrated good efficacy in HPV + HNSCC in combination with radiation [146].

Recent studies suggest targeting alternative components of the ubiquitin system may have clinical benefit in HPV+ cancers. Given the recent study of USP7 as a deubiquitinating enzyme of HPV16 E7 and the promise shown by the highly specific USP7 inhibitors in in vivo models [147], these USP7 inhibitors could have therapeutic potential in HPV+ cancers. However, as another study demonstrated that USP7 levels are decreased in HPV + HNSCC [103], the clinical utility of USP7 inhibitors in these cancers is unclear and requires further investigation. Additionally, USP14 has been shown to play an important role in cervical cancer and both HPV+ and HPV- HNSCC, and small molecule inhibitors have entered early phase clinical trials, albeit with limited success [148, 149]. Together, these studies highlight potential cellular targets that could be of potential use in HPV+ cancers. Several of these have small molecule inhibitors in preclinical or clinical development, furthering their potential as therapeutic options in these cancers.

## Future directions

Accumulating evidence suggests that the manipulation of protein ubiquitination and ubiquitin-like protein conjugation may play an important role in HPV-mediated transformation, providing an insight into the strategies HPV uses in initiating and promoting tumour development. Whilst many interactions between the HPV oncoproteins and the ubiquitin machinery and the UPS have been identified, many aspects still remain unclear. Many of these interactions have only been confirmed for one HPV type in one or two cell lines. These studies should be confirmed in multiple cell lines and cell lines with endogenous HPV oncoprotein expression to confirm their functions. Furthermore, many of these studies have been conducted in cell-lines overexpressing or depleting the protein of interest and have only been demonstrated in a single study; therefore, further studies should be carried out in HPV-driven malignancies to determine if ubiquitin and ubiquitin-like proteins are valuable clinical targets. These analyses are essential to confirm the contributory role of these interactions in HPV driven carcinogenesis. Given the increasing interest in targeting protein ubiquitination as novel cancer therapies, and the systemic targeting of ubiquitination and the UPS by tumour viruses, future studies are warranted to fill in these gaps in knowledge and potentially uncover novel therapeutic targets in HPV+ cancers. Efforts should be focused on developing novel strategies to inhibit these interactions when considering the increasing need for novel therapeutics for HPV-associated cancer.

## References

[1] Zheng ZM, Baker CC. Papillomavirus genome structure, expression, and post-transcriptional regulation. Front Biosci. 2006;11:2286–302. 16720315 10.2741/1971 PMC1472295

[2] Doorbar J, Egawa N, Griffin H, Kranjec C, Murakami I. Human papillomavirus molecular biology and disease association. Rev Med Virol. 2015;25:2–23. 25752814 10.1002/rmv.1822 PMC5024016

[3] Van Doorslaer K, Li Z, Xirasagr S, Maes P, Kaminsky D, Liou D, et al. The papillomavirus episteme: a major update to the papillomavirus sequence database. Nucleic Acids Res. 2017;45:D499–506. 28053164 10.1093/nar/gkw879 PMC5210616

[4] Graham SV. The human papillomavirus replication cycle, and its links to cancer progression: a comprehensive review. Clin Sci. 2017;131:2201–21. 10.1042/CS20160786 28798073

[5] Moody CA. Regulation of the innate immune response during the human papillomavirus life cycle. Viruses. 2022;14:1797. 36016419 10.3390/v14081797 PMC9412305

[6] Buck CB, Thompson CD, Pang Y-YS, Lowy DR, Schiller JT. Maturation of papillomavirus capsids. J Virol. 2005;79:2839–46. 15709003 10.1128/JVI.79.5.2839-2846.2005 PMC548454

[7] Bryan JT, Brown DR. Transmission of human papillomavirus type 11 infection by desquamated cornified cells. Virology. 2001;281:35–42. 11222093 10.1006/viro.2000.0777

[8] de Martel C, Plummer M, Vignat J, Franceschi S. Worldwide burden of cancer attributable to HPV by site, country and HPV type. Int J Cancer. 2017;141:664–70. 28369882 10.1002/ijc.30716 PMC5520228

[9] Shanmugasundaram S, You J. Targeting persistent human papillomavirus infection. Viruses. 2017;9:229. 10.3390/v9080229 PMC5580486 28820433

[10] Scarth JA, Patterson MR, Morgan EL, Macdonald A. The human papillomavirus oncoproteins: a review of the host pathways targeted on the road to transformation. J General Virol. 2021;102:001540. 10.1099/jgv.0.001540 PMC8148304 33427604

[11] Dyson N, Guida P, Münger K, Harlow E. Homologous sequences in adenovirus E1A and human papillomavirus E7 proteins mediate interaction with the same set of cellular proteins. J Virol. 1992;66:6893–902. 1331501 10.1128/jvi.66.12.6893-6902.1992 PMC240306

[12] Klingelhutz AJ, Foster SA, McDougall JK. Telomerase activation by the E6 gene product of human papillomavirus type 16. Nature. 1996;380:79–82. 8598912 10.1038/380079a0

[13] Genther Williams SM, Disbrow GL, Schlegel R, Lee D, Threadgill DW, Lambert PF. Requirement of epidermal growth factor receptor for hyperplasia induced by E5, a high-risk human papillomavirus oncogene. Cancer Res. 2005;65:6534–42. 16061632 10.1158/0008-5472.CAN-05-0083

[14] Wasson CW, Morgan EL, Müller M, Ross RL, Hartley M, Roberts S, et al. Human papillomavirus type 18 E5 oncogene supports cell cycle progression and impairs epithelial differentiation by modulating growth factor receptor signalling during the virus life cycle. Oncotarget. 2017;8:103581–600. 29262586 10.18632/oncotarget.21658 PMC5732752

[15] Sun T, Liu Z, Yang Q. The role of ubiquitination and deubiquitination in cancer metabolism. Mol Cancer. 2020;19:146. 33004065 10.1186/s12943-020-01262-x PMC7529510

[16] Callis J, Pollmann L, Shanklin J, Wettern M, Vierstra RD. Sequence of a cDNA from *Chlamydomonas reinhardii* encoding a ubiquitin 52 amino acid extension protein. Nucleic Acids Res. 1989;17:8377. 2554258 10.1093/nar/17.20.8377 PMC334983

[17] Vijay-kumar S, Bugg CE, Cook WJ. Structure of ubiquitin refined at 1.8 Å resolution. J Mol Biol. 1987;194:531–44. 3041007 10.1016/0022-2836(87)90679-6

[18] Walczak H, Iwai K, Dikic I. Generation and physiological roles of linear ubiquitin chains. BMC Biol. 2012;10:23. 22420778 10.1186/1741-7007-10-23 PMC3305636

[19] Cruz Walma DA, Chen Z, Bullock AN, Yamada KM. Ubiquitin ligases: guardians of mammalian development. Nat Rev Mol Cell Biol. 2022;23:350–67. 35079164 10.1038/s41580-021-00448-5

[20] Swatek KN, Komander D. Ubiquitin modifications. Cell Res. 2016;26:399–422. 27012465 10.1038/cr.2016.39 PMC4822133

[21] Ikeda F, Dikic I. Atypical ubiquitin chains: new molecular signals. EMBO Rep. 2008;9:536–42. 18516089 10.1038/embor.2008.93 PMC2427391

[22] Ikeda F, Crosetto N, Dikic I. What determines the specificity and outcomes of ubiquitin signaling? Cell. 2010;143:677–81. 21111228 10.1016/j.cell.2010.10.026

[23] Sakamaki J, Mizushima N. Ubiquitination of non-protein substrates. Trends Cell Biol. 2023;33:991–1003. 37120410 10.1016/j.tcb.2023.03.014

[24] Kim JH. Deubiquitinating enzymes as cellular regulators. J Biochem. 2003;134:9–18. 12944365 10.1093/jb/mvg107

[25] Ciehanover A, Hod Y, Hershko A. A heat-stable polypeptide component of an ATP-dependent proteolytic system from reticulocytes. Biochem Biophys Res Commun. 1978;81:1100–5. 666810 10.1016/0006-291x(78)91249-4

[26] Ciechanover A, Heller H, Katz-Etzion R, Hershko A. Activation of the heat-stable polypeptide of the ATP-dependent proteolytic system. Proc Natl Acad Sci USA. 1981;78:761–5. 6262770 10.1073/pnas.78.2.761 PMC319882

[27] Ciechanover A, Schwartz AL. The ubiquitin-proteasome pathway: the complexity and myriad functions of proteins death. Proc Natl Acad Sci USA. 1998;95:2727–30. 9501156 10.1073/pnas.95.6.2727 PMC34259

[28] Finley D. Recognition and processing of ubiquitin-protein conjugates by the proteasome. Annu Rev Biochem. 2009;78:477–513. 19489727 10.1146/annurev.biochem.78.081507.101607 PMC3431160

[29] Zou T, Lin Z. The involvement of ubiquitination machinery in cell cycle regulation and cancer progression. Int J Mol Sci. 2021;22:5754. 10.3390/ijms22115754 PMC8198665 34072267

[30] Celebi G, Kesim H, Ozer E, Kutlu O. The effect of dysfunctional ubiquitin enzymes in the pathogenesis of most common diseases. Int J Mol Sci. 2020;21:6335. 32882786 10.3390/ijms21176335 PMC7503467

[31] Wang T, Jiang J, Zhang X, Ke X, Qu Y. Ubiquitin-like modification dependent proteasomal degradation and disease therapy. Trends Mol Med. 2024;30:1061–75. 38851992 10.1016/j.molmed.2024.05.005

[32] Petroski MD, Deshaies RJ. Function and regulation of cullin–RING ubiquitin ligases. Nat Rev Mol Cell Biol. 2005;6:9–20. 15688063 10.1038/nrm1547

[33] Celen AB, Sahin U. Sumoylation on its 25th anniversary: mechanisms, pathology, and emerging concepts. FEBS J. 2020;287:3110–40. 32255256 10.1111/febs.15319

[34] Perng Y-C, Lenschow DJ. ISG15 in antiviral immunity and beyond. Nat Rev Microbiol. 2018;16:423–39. 29769653 10.1038/s41579-018-0020-5 PMC7097117

[35] Aichem A, Groettrup M. The ubiquitin-like modifier FAT10 – much more than a proteasome-targeting signal. J Cell Sci. 2020;133:jcs246041. 32719056 10.1242/jcs.246041

[36] Mizushima N. The ATG conjugation systems in autophagy. Curr Opin Cell Biol. 2020;63:1–0. 10.1016/j.ceb.2019.12.001 31901645

[37] Zhang X, Chen X-L. The emerging roles of ubiquitin-like protein Urm1 in eukaryotes. Cell Signal. 2021;81:109946. 33548388 10.1016/j.cellsig.2021.109946

[38] Wang X, Xu X, Wang Z. The post-translational role of UFMylation in physiology and disease. Cells. 2023;12:2543. 10.3390/cells12212543 PMC10648299 37947621

[39] Luo H. Interplay between the virus and the ubiquitin–proteasome system: molecular mechanism of viral pathogenesis. Curr Opin Virol. 2016;17:1–10. 26426962 10.1016/j.coviro.2015.09.005 PMC7102833

[40] Senft D, Qi J, Ronai ZA. Ubiquitin ligases in oncogenic transformation and cancer therapy. Nat Rev Cancer. 2018;18:69–88. 29242641 10.1038/nrc.2017.105 PMC6054770

[41] Scheffner M, Huibregtse JM, Vierstra RD, Howley PM. The HPV-16 E6 and E6-AP complex functions as a ubiquitin-protein ligase in the ubiquitination of p53. Cell. 1993;75:495–505. 8221889 10.1016/0092-8674(93)90384-3

[42] Giarrè M, Caldeira S, Malanchi I, Ciccolini F, Leão MJ, Tommasino M. Induction of pRb degradation by the human papillomavirus type 16 E7 protein is essential to efficiently overcome p16 ^INK4a^ -imposed G _1_ cell cycle arrest. J Virol. 2001;75:4705–12. 11312342 10.1128/JVI.75.10.4705-4712.2001 PMC114225

[43] Đukić A, Lulić L, Thomas M. HPV oncoproteins and the ubiquitin proteasome system: a signature of malignancy? Pathogens. 2020;9:133. 32085533 10.3390/pathogens9020133 PMC7168213

[44] Pei Y, Robertson ES. The central role of the ubiquitin–proteasome system in EBV-mediated oncogenesis. Cancers (Basel). 2022;14:611. 10.3390/cancers14030611 PMC8833352 35158879

[45] Dybas JM, Herrmann C, Weitzman MD. Ubiquitination at the interface of tumor viruses and DNA damage responses. Curr Opin Virol. 2018;32:40–7. 30261451 10.1016/j.coviro.2018.08.017 PMC6263849

[46] Huibregtse JM, Scheffner M, Howley PM. Cloning and expression of the cDNA for E6-AP, a protein that mediates the interaction of the human papillomavirus E6 incoprotein with p53. Mol Cell Biol. 1993;13:775–84. 8380895 10.1128/mcb.13.2.775-784.1993 PMC358960

[47] Brimer N, Lyons C, Vande Pol SB. Association of E6AP (UBE3A) with human papillomavirus type 11 E6 protein. Virology. 2007;358:303–10. 17023019 10.1016/j.virol.2006.08.038 PMC1892534

[48] Accardi R, Rubino R, Scalise M, Gheit T, Shahzad N, Thomas M, et al. E6 and E7 from human papillomavirus type 16 cooperate to target the PDZ protein Na/H exchange regulatory factor 1. J Virol. 2011;85:8208–16. 21680517 10.1128/JVI.00114-11 PMC3147992

[49] Drews CM, Case S, Vande Pol SB. E6 proteins from high-risk HPV, low-risk HPV, and animal papillomaviruses activate the Wnt/β-catenin pathway through E6AP-dependent degradation of NHERF1. PLoS Pathog. 2019;15:e1007575. 31002735 10.1371/journal.ppat.1007575 PMC6493770

[50] Cosper PF, Hrycyniak LC, Paracha M, Lee DL, Wan J, Jones K, et al. HPV16 E6 induces chromosomal instability due to polar chromosomes caused by E6AP-dependent degradation of the mitotic kinesin CENP-E. Proc Natl Acad Sci USA. 2023;120:e2216700120. 10.1073/pnas.2216700120 PMC10083562 36989302

[51] Shearer RF, Iconomou M, Watts CKW, Saunders DN. Functional roles of the E3 ubiquitin ligase UBR5 in cancer. Mol Cancer Res. 2015;13:1523–32. 26464214 10.1158/1541-7786.MCR-15-0383

[52] Schleicher EM, Dhoonmoon A, Jackson LM, Khatib JB, Nicolae CM, Moldovan GL. The TIP60-ATM axis regulates replication fork stability in BRCA-deficient cells. Oncogenesis. 2022;11:33. 35717336 10.1038/s41389-022-00410-w PMC9206655

[53] Jha S, Pol SV, Banerjee NS, Dutta AB, Chow LT, Dutta A. Destabilization of TIP60 by human papillomavirus E6 results in attenuation of TIP60-dependent transcriptional regulation and apoptotic pathway. Mol Cell. 2010;38:700–11. 20542002 10.1016/j.molcel.2010.05.020 PMC2886028

[54] Subbaiah VK, Zhang Y, Rajagopalan D, Abdullah LN, Yeo-Teh NS, Tomaić V, et al. E3 ligase EDD1/UBR5 is utilized by the HPV E6 oncogene to destabilize tumor suppressor TIP60. Oncogene. 2016;35:2062–74. 26234678 10.1038/onc.2015.268

[55] Tomaic V, Pim D, Thomas M, Massimi P, Myers MP, Banks L. Regulation of the human papillomavirus type 18 E6/E6AP ubiquitin ligase complex by the HECT domain-containing protein EDD. J Virol. 2011;85:3120–7. 21228227 10.1128/JVI.02004-10 PMC3067830

[56] Khalil MI, Yang C, Vu L, Chadha S, Nabors H, Welbon C, et al. HPV upregulates MARCHF8 ubiquitin ligase and inhibits apoptosis by degrading the death receptors in head and neck cancer. PLoS Pathog. 2023;19:e1011171. 36867660 10.1371/journal.ppat.1011171 PMC10016708

[57] Münger K, Werness BA, Dyson N, Phelps WC, Harlow E, Howley PM. Complex formation of human papillomavirus E7 proteins with the retinoblastoma tumor suppressor gene product. EMBO J. 1989;8:4099–105. 2556261 10.1002/j.1460-2075.1989.tb08594.x PMC401588

[58] Dyson N, Howley PM, Münger K, Harlow E. the human papilloma virus-16 E7 oncoprotein is able to bind to the retinoblastoma gene product. Science. 1989;243:934–7. 2537532 10.1126/science.2537532

[59] Huh K, Zhou X, Hayakawa H, Cho JY, Libermann TA, Jin J, et al. Human papillomavirus type 16 E7 oncoprotein associates with the cullin 2 ubiquitin ligase complex, which contributes to degradation of the retinoblastoma tumor suppressor. J Virol. 2007;81:9737–47. 17609271 10.1128/JVI.00881-07 PMC2045412

[60] White EA, Sowa ME, Tan MJ, Jeudy S, Hayes SD, Santha S, et al. Systematic identification of interactions between host cell proteins and E7 oncoproteins from diverse human papillomaviruses. Proc Natl Acad Sci USA. 2012;109:E260–7. 22232672 10.1073/pnas.1116776109 PMC3277141

[61] Fischer M, Uxa S, Stanko C, Magin TM, Engeland K. Human papilloma virus E7 oncoprotein abrogates the p53-p21-DREAM pathway. Sci Rep. 2017;7:2603. 28572607 10.1038/s41598-017-02831-9 PMC5453983

[62] Nouel J, White EA. ZER1 contributes to the carcinogenic activity of high-risk HPV E7 proteins. Mbio. 2022;13:e02033-22. 10.1128/mbio.02033-22 PMC9765665 36346242

[63] Todorovic B, Hung K, Massimi P, Avvakumov N, Dick FA, Shaw GS, et al. Conserved Region 3 of Human Papillomavirus 16 E7 Contributes to Deregulation of the Retinoblastoma Tumor Suppressor. J Virol. 2012;86:13313–23. 23015707 10.1128/JVI.01637-12 PMC3503127

[64] Westrich JA, Warren CJ, Klausner MJ, Guo K, Liu CW, Santiago ML, et al. Human papillomavirus 16 E7 stabilizes APOBEC3a protein by inhibiting cullin 2-dependent protein degradation. J Virol. 2018;92:10–128. 10.1128/JVI.01318-17 PMC5972886 29367246

[65] Huh KW, DeMasi J, Ogawa H, Nakatani Y, Howley PM, Münger K. Association of the human papillomavirus type 16 E7 oncoprotein with the 600-kDa retinoblastoma protein-associated factor, p600. Proc Natl Acad Sci USA. 2005;102:11492–7. 16061792 10.1073/pnas.0505337102 PMC1182135

[66] White EA, Münger K, Howley PM. High-risk human papillomavirus E7 proteins target PTPN14 for degradation. mBio. 2016;7:e01530–16. 27651363 10.1128/mBio.01530-16 PMC5030362

[67] Szalmás A, Tomaić V, Basukala O, Massimi P, Mittal S, Kónya J, et al. The PTPN14 tumor suppressor is a degradation target of human papillomavirus E7. J Virol. 2017;91:10–128. 10.1128/JVI.00057-17 PMC5355602 28100625

[68] Liu X, Yang N, Figel SA, Wilson KE, Morrison CD, Gelman IH, et al. PTPN14 interacts with and negatively regulates the oncogenic function of YAP. Oncogene. 2013;32:1266–73. 22525271 10.1038/onc.2012.147 PMC4402938

[69] Wilson KE, Li YW, Yang N, Shen H, Orillion AR, Zhang J. PTPN14 forms a complex with Kibra and LATS1 proteins and negatively regulates the YAP oncogenic function. J Biol Chem. 2014;289:23693–700. 25023289 10.1074/jbc.M113.534701 PMC4156080

[70] He C, Mao D, Hua G, Lv X, Chen X, Angeletti PC, et al. The Hippo/YAP pathway interacts with EGFR signaling and HPV oncoproteins to regulate cervical cancer progression. EMBO Mol Med. 2015;7:1426–49. 26417066 10.15252/emmm.201404976 PMC4644376

[71] Hatterschide J, Bohidar AE, Grace M, Nulton TJ, Kim HW, Windle B, et al. PTPN14 degradation by high-risk human papillomavirus E7 limits keratinocyte differentiation and contributes to HPV-mediated oncogenesis. Proc Natl Acad Sci USA. 2019;116:7033–42. 30894485 10.1073/pnas.1819534116 PMC6452706

[72] Morgan EL, Patterson MR, Ryder EL, Lee SY, Wasson CW, Harper KL, et al. MicroRNA-18a targeting of the STK4/MST1 tumour suppressor is necessary for transformation in HPV positive cervical cancer. PLoS Pathog. 2020;16:e1008624. 32555725 10.1371/journal.ppat.1008624 PMC7326282

[73] Patterson MR, Cogan JA, Cassidy R, Theobald DA, Wang M, Scarth JA, et al. The Hippo pathway transcription factors YAP and TAZ play HPV-type dependent roles in cervical cancer. Nat Commun. 2024;15:5809. 38987584 10.1038/s41467-024-49965-9 PMC11237029

[74] Munger K, White EA. What are the essential determinants of human papillomavirus carcinogenesis? mBio. 2024;15:e00462-24. 10.1128/mbio.00462-24 PMC11558995 39365046

[75] Sitz J, Blanchet SA, Gameiro SF, Biquand E, Morgan TM, Galloy M, et al. Human papillomavirus E7 oncoprotein targets RNF168 to hijack the host DNA damage response. Proc Natl Acad Sci USA. 2019;116:19552–62. 31501315 10.1073/pnas.1906102116 PMC6765264

[76] Cao J, Ge M-H, Ling Z-Q. Fbxw7 tumor suppressor: a vital regulator contributes to human tumorigenesis. Medicine. 2016;95:e2496. 26886596 10.1097/MD.0000000000002496 PMC4998596

[77] Spaans VM, Mahendra IN, Purwoto G, Trietsch MD, Osse M, Ter Haar N, et al. The landscape of somatic mutations in Indonesian cervical cancer is predominated by the PI3K pathway. Gynecol Oncol. 2018;148:189–96. 29113722 10.1016/j.ygyno.2017.10.009

[78] Ojesina AI, Lichtenstein L, Freeman SS, Pedamallu CS, Imaz-Rosshandler I, Pugh TJ, et al. Landscape of genomic alterations in cervical carcinomas. Nature. 2014;506:371–5. 24390348 10.1038/nature12881 PMC4161954

[79] Zhu X, Jamshed S, Zou J, Azar A, Meng X, Bathini V, et al. Molecular and immunophenotypic characterization of anal squamous cell carcinoma reveals distinct clinicopathologic groups associated with HPV and TP53 mutation status. Mod Pathol. 2021;34:1017–30. 33483624 10.1038/s41379-020-00729-y

[80] Williams EA, Werth AJ, Sharaf R, Montesion M, Sokol ES, Pavlick DC, et al. Vulvar squamous cell carcinoma: comprehensive genomic profiling of HPV+ versus HPV– forms reveals distinct sets of potentially actionable molecular targets. JCO Precis Oncol. 2020;4:647–61. 10.1200/PO.19.00406 PMC7446361 32923875

[81] Lechner M, Frampton GM, Fenton T, Feber A, Palmer G, Jay A, et al. Targeted next-generation sequencing of head and neck squamous cell carcinoma identifies novel genetic alterations in HPV+ and HPV- tumors. Genome Med. 2013;5:49. 23718828 10.1186/gm453 PMC4064312

[82] Liu F, Zou Y, Wang F, Yang B, Zhang Z, Luo Y, et al. *FBXW7* mutations promote cell proliferation, migration, and invasion in cervical cancer. Genet Test Mol Biomark. 2019;23:409–17. 10.1089/gtmb.2018.0278 31161818

[83] Cancer Genome Atlas Network. Comprehensive genomic characterization of head and neck squamous cell carcinomas. Nature. 2015;517:576–82. 25631445 10.1038/nature14129 PMC4311405

[84] Hajek M, Sewell A, Kaech S, Burtness B, Yarbrough WG, Issaeva N. TRAF3/CYLD mutations identify a distinct subset of human papillomavirus-associated head and neck squamous cell carcinoma. Cancer. 2017;123:1778–90. 28295222 10.1002/cncr.30570 PMC5419871

[85] Zarnegar BJ, Wang Y, Mahoney DJ, Dempsey PW, Cheung HH, He J, et al. Noncanonical NF-κB activation requires coordinated assembly of a regulatory complex of the adaptors cIAP1, cIAP2, TRAF2 and TRAF3 and the kinase NIK. Nat Immunol. 2008;9:1371–8. 18997794 10.1038/ni.1676 PMC2676931

[86] Zhang J, Chen T, Yang X, Cheng H, Späth SS, Clavijo PE, et al. Attenuated TRAF3 fosters activation of alternative NF-κB and reduced expression of antiviral interferon, TP53, and RB to promote HPV-positive head and neck cancers. Cancer Res. 2018;78:4613–26. 29921694 10.1158/0008-5472.CAN-17-0642 PMC7983169

[87] Chen D, Ning Z, Chen H, Lu C, Liu X, Xia T, et al. An integrative pan-cancer analysis of biological and clinical impacts underlying ubiquitin-specific-processing proteases. Oncogene. 2020;39:587–602. 31511647 10.1038/s41388-019-1002-4

[88] Zheng L-L, Wang L-T, Pang Y-W, Sun L-P, Shi L. Recent advances in the development of deubiquitinases inhibitors as antitumor agents. Eur J Med Chem. 2024;266:116161. 38262120 10.1016/j.ejmech.2024.116161

[89] An J, Mo D, Liu H, Veena MS, Srivatsan ES, Massoumi R, et al. Inactivation of the CYLD deubiquitinase by HPV E6 mediates hypoxia-induced NF-kappaB activation. Cancer Cell. 2008;14:394–407. 18977328 10.1016/j.ccr.2008.10.007 PMC2651888

[90] Tilborghs S, Corthouts J, Verhoeven Y, Arias D, Rolfo C, Trinh XB, et al. The role of nuclear factor-kappa B signaling in human cervical cancer. Crit Rev Oncol Hematol. 2017;120:141–50. 29198328 10.1016/j.critrevonc.2017.11.001

[91] Hu Z, Viswanathan R, Cheng H, Chen J, Yang X, Huynh A, et al. Inhibiting WEE1 and IKK-RELA crosstalk overcomes TNFα resistance in head and neck cancers. Mol Cancer Res. 2022;20:867–82. 35176168 10.1158/1541-7786.MCR-21-0624 PMC9177594

[92] Morgan EL, Macdonald A. Autocrine STAT3 activation in HPV positive cervical cancer through a virus-driven Rac1—NFκB—IL-6 signalling axis. PLoS Pathog. 2019;15:e1007835. 31226168 10.1371/journal.ppat.1007835 PMC6608985

[93] Chen Z, Viswanathan R, Morgan EL, Jeon J & Van Waes C Proinflammatory Signaling Pathways and Genomic Signatures in Head and Neck Cancers. in *Early Detection and Treatment of Head & Neck Cancers* 143-84 (Springer International Publishing, Cham, 2021).

[94] Kiran S, Dar A, Singh SK, Lee KY, Dutta A. The deubiquitinase USP46 is essential for proliferation and tumor growth of HPV-transformed cancers. Mol Cell. 2018;72:823–35.e5. 30415951 10.1016/j.molcel.2018.09.019 PMC6294304

[95] Kiran S, Wilson B, Saha S, Graff JA, Dutta A. HPVE6-USP46 mediated Cdt2 stabilization reduces Set8 mediated H4K20-methylation to induce gene expression changes. Cancers (Basel). 2021;14:30. 35008200 10.3390/cancers14010030 PMC8750077

[96] Morgan EL, Patterson MR, Barba-Moreno D, Scarth JA, Wilson A, Macdonald A. The deubiquitinase (DUB) USP13 promotes Mcl-1 stabilisation in cervical cancer. Oncogene. 2021;40:2112–29. 33627786 10.1038/s41388-021-01679-8 PMC7979541

[97] Bhatt S, Pioso MS, Olesinski EA, Yilma B, Ryan JA, Mashaka T, et al. Reduced mitochondrial apoptotic priming drives resistance to bh3 mimetics in acute myeloid leukemia. Cancer Cell. 2020;38:872–90.e6. 33217342 10.1016/j.ccell.2020.10.010 PMC7988687

[98] Townsend PA, Kozhevnikova MV, Cexus ONF, Zamyatnin AA, Soond SM. BH3-mimetics: recent developments in cancer therapy. J Exp Clin Cancer Res. 2021;40:355. 34753495 10.1186/s13046-021-02157-5 PMC8576916

[99] Wu L, Lin Y, Feng J, Qi Y, Wang X, Lin Q, et al. The deubiquitinating enzyme OTUD1 antagonizes BH3-mimetic inhibitor induced cell death through regulating the stability of the MCL1 protein. Cancer Cell Int. 2019;19:222. 31467488 10.1186/s12935-019-0936-5 PMC6712616

[100] Wu X, Luo Q, Zhao P, Chang W, Wang Y, Shu T, et al. JOSD1 inhibits mitochondrial apoptotic signalling to drive acquired chemoresistance in gynaecological cancer by stabilizing MCL1. Cell Death Differ. 2020;27:55–70. 31043700 10.1038/s41418-019-0339-0 PMC7206032

[101] Dok R, Kalev P, Van Limbergen EJ, Asbagh LA, Vázquez I, Hauben E, et al. p16INK4a Impairs Homologous Recombination–Mediated DNA Repair in Human Papillomavirus–Positive Head and Neck Tumors. Cancer Res. 2014;74:1739–51. 24473065 10.1158/0008-5472.CAN-13-2479

[102] Robles SJ, Adami GR. Agents that cause DNA double strand breaks lead to p16INK4a enrichment and the premature senescence of normal fibroblasts. Oncogene. 1998;16:1113–23. 9528853 10.1038/sj.onc.1201862

[103] Molkentine DP, Molkentine JM, Bridges KA, Valdecanas DR, Dhawan A, Bahri R, et al. p16 represses DNA damage repair via a novel ubiquitin-dependent signaling cascade. Cancer Res. 2022;82:916–28. 34965932 10.1158/0008-5472.CAN-21-2101 PMC9136619

[104] Stewart D. Ubiquitination and proteasome degradation of the E6 proteins of human papillomavirus types 11 and 18. J Gen Virol. 2004;85:1419–26. 15166424 10.1099/vir.0.19679-0

[105] Selvey LA, Dunn LA, Tindle RW, Park DS, Frazer IH. Human papillomavirus (HPV) type 18 E7 protein is a short-lived steroid-inducible phosphoprotein in HPV-transformed cell lines. J Gen Virol. 1994;75:1647–53. 8021595 10.1099/0022-1317-75-7-1647

[106] Ansari T, Brimer N, Vande Pol SB. Peptide interactions stabilize and restructure human papillomavirus type 16 E6 to interact with p53. J Virol. 2012;86:11386–91. 22896608 10.1128/JVI.01236-12 PMC3457172

[107] García-Alai MM, Dantur KI, Smal C, Pietrasanta L, de Prat-Gay G. High-Risk HPV E6 oncoproteins assemble into large oligomers that allow localization of endogenous species in prototypic HPV-transformed cell lines. Biochemistry. 2007;46:341–9. 17209544 10.1021/bi602457q

[108] Clemens KE, Bren R, Gyuris J, Münger K. Dimerization of the human papillomavirus E7 oncoproteinin vivo. Virology. 1995;214:289–93. 8525630 10.1006/viro.1995.9926

[109] Ajiro M, Zhen ZM. E6^E7, a novel splice isoform protein of human papillomavirus 16, stabilizes viral E6 and E7 oncoproteins via HSP90 and GRP78. mBio. 2015;6:10–128. 10.1128/mBio.02068-14 PMC4337564 25691589

[110] Li S, Hong X, Wei Z, Xie M, Li W, Liu G, et al. Ubiquitination of the HPV oncoprotein E6 is critical for E6/E6AP-mediated p53 degradation. Front Microbiol. 2019;10:2483. 10.3389/fmicb.2019.02483 PMC6842930 31749782

[111] Tomaić V, Pim D, Banks L. The stability of the human papillomavirus E6 oncoprotein is E6AP dependent. Virology. 2009;393:7–10. 19700180 10.1016/j.virol.2009.07.029

[112] Thatte J, Banks L. Human papillomavirus 16 (HPV-16), HPV-18, and HPV-31 E6 override the normal phosphoregulation of E6AP enzymatic activity. J Virol. 2017;91:10–128. 10.1128/JVI.01390-17 PMC5660492 28835500

[113] Vats A, Braga L, Kavcic N, Massimi P, Schneider E, Giacca M, et al. Regulation of human papillomavirus E6 oncoprotein function via a novel ubiquitin ligase FBXO4. mBio. 2025;16:e02783-24. 10.1128/mbio.02783-24 PMC11796345 39688415

[114] Reinstein E, Scheffner M, Oren M, Ciechanover A, Schwartz A. Degradation of the E7 human papillomavirus oncoprotein by the ubiquitin-proteasome system: targeting via ubiquitination of the N-terminal residue. Oncogene. 2000;19:5944–50. 11127826 10.1038/sj.onc.1203989

[115] Ciechanover A. N-terminal ubiquitination: more protein substrates join in. Trends Cell Biol. 2004;14:103–6. 15055197 10.1016/j.tcb.2004.01.004

[116] Oh K-J, et al. The papillomavirus E7 oncoprotein is ubiquitinated by UbcH7 and Cullin 1- and Skp2-containing E3 ligase. J Virol. 2004;78:5338–46. 15113913 10.1128/JVI.78.10.5338-5346.2004 PMC400333

[117] Khalil MI, Yang C, Vu L, Chadha S, Nabors H, James CD, et al. The membrane-associated ubiquitin ligase MARCHF8 stabilizes the human papillomavirus oncoprotein E7 by degrading CUL1 and UBE2L3 in head and neck cancer. J Virol. 2024;98:e01726-23. 10.1128/jvi.01726-23 PMC10878100 38226814

[118] Kamio M, Yoshida T, Ogata H, Douchi T, Nagata Y, Inoue M, et al. SOC1 inhibits HPV-E7-mediated transformation by inducing degradation of E7 protein. Oncogene. 2004;23:3107–15. 15021916 10.1038/sj.onc.1207453

[119] Hayman TJ, Baro M, MacNeil T, Phoomak C, Aung TN, Cui W, et al. STING enhances cell death through regulation of reactive oxygen species and DNA damage. Nat Commun. 2021;12:2327. 33875663 10.1038/s41467-021-22572-8 PMC8055995

[120] Luo X, Donnelly CR, Gong W, Heath BR, Hao Y, Donnelly LA, et al. HPV16 drives cancer immune escape via NLRX1-mediated degradation of STING. J Clin Investig. 2020;130:1635–52. 31874109 10.1172/JCI129497 PMC7108911

[121] Huang X, Huo L, Xiao B, Ouyang Y, Chen F, Li J, et al. Activating STING/TBK1 suppresses tumor growth via degrading HPV16/18 E7 oncoproteins in cervical cancer. Cell Death Differ. 2024;31:78–89. 38007552 10.1038/s41418-023-01242-w PMC10781763

[122] Vats A, Trejo-Cerro O, Massimi P, Banks L. Regulation of HPV E7 stability by E6-associated protein (E6AP). J Virol. 2022;96:e00663-22. 10.1128/jvi.00663-22 PMC9400497 35916535

[123] Tomaić V, Ganti K, Pim D, Bauer C, Blattner C, Banks L. Interaction of HPV E6 oncoproteins with specific proteasomal subunits. Virology. 2013;446:389–96. 24074603 10.1016/j.virol.2013.08.016

[124] Berezutskaya E, Bagchi S. The human papillomavirus E7 oncoprotein functionally interacts with the S4 subunit of the 26 S proteasome. J Biol Chem. 1997;272:30135–40. 9374493 10.1074/jbc.272.48.30135

[125] Vos RM, Altreuter J, White EA, Howley PM. The ubiquitin-specific peptidase USP15 regulates human papillomavirus type 16 E6 protein stability. J Virol. 2009;83:8885–92. 19553310 10.1128/JVI.00605-09 PMC2738190

[126] Yaginuma Y, Yoshimoto M, Tokuda A. USP15 inhibits HPV16 E6 degradation and catalytically inactive USP15 has reduced inhibitory activity. Acta Virol. 2018;62:147–56. 29895155 10.4149/av_2018_204

[127] Chiang C, Pauli EK, Biryukov J, Feister KF, Meng M, White EA, et al. The human papillomavirus E6 oncoprotein targets USP15 and TRIM25 To suppress RIG-I-mediated innate immune signaling. J Virol. 2018;92:10–128. 10.1128/JVI.01737-17 PMC5827370 29263274

[128] Poirson J, Biquand E, Straub ML, Cassonnet P, Nominé Y, Jones L, et al. Mapping the interactome of HPV E6 and E7 oncoproteins with the ubiquitin‐proteasome system. FEBS J. 2017;284:3171–201. 28786561 10.1111/febs.14193

[129] Lin CH, Chang HS, Yu WCY. USP11 stabilizes HPV-16E7 and further modulates the E7 biological activity. J Biol Chem. 2008;283:15681–8. 18408009 10.1074/jbc.M708278200 PMC3259633

[130] Xia C, Xiao C, Luk HY, Chan PKS, Boon SS. The ubiquitin specific protease 7 stabilizes HPV16E7 to promote HPV-mediated carcinogenesis. Cell Mol Life Sci. 2023;80:278. 37682346 10.1007/s00018-023-04941-2 PMC11072444

[131] Bojagora A, Saridakis V. USP7 manipulation by viral proteins. Virus Res. 2020;286:198076. 32603670 10.1016/j.virusres.2020.198076

[132] Hochstrasser M. Origin and function of ubiquitin-like proteins. Nature. 2009;458:422–9. 19325621 10.1038/nature07958 PMC2819001

[133] Zhang Q, Qiao L, Wang X, Ding C, Chen JJ. UHRF1 epigenetically down-regulates UbcH8 to inhibit apoptosis in cervical cancer cells. Cell Cycle. 2018;17:300–8. 29157076 10.1080/15384101.2017.1403686 PMC5914733

[134] Tao P, Sun L, Sun Y, Wang Y, Yang Y, Yang B, et al. ISG15 is associated with cervical cancer development. Oncol Lett. 2022;24:380. 36238852 10.3892/ol.2022.13500 PMC9494601

[135] Mao H, Wang M, Cao B, Zhou H, Zhang Z, Mao X. Interferon-stimulated gene 15 induces cancer cell death by suppressing the NF-κB signaling pathway. Oncotarget. 2016;7:70143–51. 27659523 10.18632/oncotarget.12160 PMC5342541

[136] Fan Y, Li X, Zhang L, Zong Z, Wang F, Huang J, et al. SUMOylation in viral replication and antiviral defense. Adv Sci. 2022;9:2104126. 10.1002/advs.202104126 PMC8895153 35060688

[137] Heaton PR, Deyrieux AF, Bian X-L, Wilson VG. HPV E6 proteins target Ubc9, the SUMO conjugating enzyme. Virus Res. 2011;158:199–208. 21510985 10.1016/j.virusres.2011.04.001 PMC3103646

[138] Mattoscio D, Casadio C, Miccolo C, Maffini F, Raimondi A, Tacchetti C, et al. Autophagy regulates UBC9 levels during viral-mediated tumorigenesis. PLoS Pathog. 2017;13:e1006262. 28253371 10.1371/journal.ppat.1006262 PMC5349695

[139] Mattoscio D, Casadio C, Fumagalli M, Sideri M, Chiocca S. The SUMO conjugating enzyme UBC9 as a biomarker for cervical HPV infections. Ecancermedicalscience. 2015;9:534. 26015803 10.3332/ecancer.2015.534 PMC4435752

[140] Baek K, Scott DC, Schulman BA. NEDD8 and ubiquitin ligation by cullin-RING E3 ligases. Curr Opin Struct Biol. 2021;67:101–9. 33160249 10.1016/j.sbi.2020.10.007 PMC8096640

[141] Lin WC, Kuo KL, Shi CS, Wu JT, Hsieh JT, Chang HC, et al. MLN4924, a Novel NEDD8-activating enzyme inhibitor, exhibits antitumor activity and enhances cisplatin-induced cytotoxicity in human cervical carcinoma: in vitro and in vivo study. Am J Cancer Res. 2015;5:3350–62. 26807316 PMC4697682

[142] Wertz IE, Wang X. From discovery to bedside: targeting the ubiquitin system. Cell Chem Biol. 2019;26:156–77. 30554913 10.1016/j.chembiol.2018.10.022

[143] Montagut AM, Armengol M, de Pablo GG, Estrada-Tejedor R, Borrell JI, Roué G. Recent advances in the pharmacological targeting of ubiquitin-regulating enzymes in cancer. Semin Cell Dev Biol. 2022;132:213–29. 35184940 10.1016/j.semcdb.2022.02.007

[144] Van Waes C, Chang AA, Lebowitz PF, Druzgal CH, Chen Z, Elsayed YA, et al. Inhibition of nuclear factor-κB and target genes during combined therapy with proteasome inhibitor bortezomib and reirradiation in patients with recurrent head-and-neck squamous cell carcinoma. Int J Radiat Oncol*Biol*Phys. 2005;63:1400–12. 10.1016/j.ijrobp.2005.05.007 16005577

[145] Silke J, Meier P. Inhibitor of apoptosis (IAP) proteins–modulators of cell death and inflammation. Cold Spring Harb Perspect Biol. 2013;5:a008730. 10.1101/cshperspect.a008730 PMC3552501 23378585

[146] Xiao R, An Y, Ye W, Derakhshan A, Cheng H, Yang X, et al. Dual antagonist of cIAP/XIAP ASTX660 sensitizes HPV− and HPV+ head and neck cancers to TNFα, TRAIL, and radiation therapy. Clin Cancer Res. 2019;25:6463–74. 31266830 10.1158/1078-0432.CCR-18-3802 PMC6825532

[147] Turnbull AP, Ioannidis S, Krajewski WW, Pinto-Fernandez A, Heride C, Martin AC, et al. Molecular basis of USP7 inhibition by selective small-molecule inhibitors. Nature. 2017;550:481–6. 29045389 10.1038/nature24451 PMC6029662

[148] Morgan EL, Toni T, Viswanathan R, Robbins Y, Yang X, Cheng H, et al. Inhibition of USP14 promotes TNFα-induced cell death in head and neck squamous cell carcinoma (HNSCC). Cell Death Differ. 2023;30:1382–96. 37055579 10.1038/s41418-023-01144-x PMC10154301

[149] Song H, Qiu J, Hua K. USP14 promotes the proliferation of cervical cancer via upregulating β‐catenin. Environ Toxicol. 2024;39:1031–43. 38069565 10.1002/tox.23990

